# Detailed Analysis of Insulin Absorption Variability and the Tissue Response to Continuous Subcutaneous Insulin Infusion Catheter Implantation in Swine

**DOI:** 10.1089/dia.2017.0175

**Published:** 2017-11-01

**Authors:** Jasmin R. Hauzenberger, Brian R. Hipszer, Channy Loeum, Peter A. McCue, Mark DeStefano, Marc C. Torjman, Mahmut T. Kaner, Alek R. Dinesen, Inna Chervoneva, Thomas R. Pieber, Jeffrey I Joseph

**Affiliations:** ^1^ Department of Anesthesiology, Jefferson Artificial Pancreas Center, Sidney Kimmel Medical College, Thomas Jefferson University, Philadelphia, Pennsylvania.; ^2^ Division of Endocrinology and Diabetology, Department of Internal Medicine, Medical University of Graz, Graz, Austria.; ^3^ Department of Pathology, Sidney Kimmel Medical College, Thomas Jefferson University, Philadelphia, Pennsylvania.; ^4^ Animas Corporation, West Chester, Pennsylvania.; ^5^ Department of Pharmacology and Experimental Therapeutics, Sidney Kimmel Medical College, Thomas Jefferson University, Philadelphia, Pennsylvania.

**Keywords:** CSII therapy, Insulin absorption variability, Inflammatory response, Adipose tissue inflammation, Insulin pharmacokinetics, CSII catheter

## Abstract

***Background:*** Worldwide, ∼1 million people manage their type 1 diabetes with an insulin pump and a continuous subcutaneous insulin infusion (CSII) catheter. Patients routinely insert a new catheter every 2–3 days due to increasing variability of insulin absorption over time. Catheter insertion and maintenance damage capillaries, lymphatics, cells, and connective tissue leading to an acute inflammatory response.

***Methods:*** We compared an investigational CSII catheter (IC) and a commercial CSII catheter (CC) regarding insulin absorption pharmacokinetics (PK) and tissue inflammation. The two different catheter designs were implanted into the subcutaneous tissue of six swine for 5 days. Insulin boluses were given on days 1, 3, and 5 of wear-time to assess PK. Tissue around catheters was excised and stained to visualize inflammation and morphological changes of adjacent tissue.

***Results:*** Insulin absorption was better when infused through a CC with highest *C*_max_ and fastest *t*_max_ values on day 5 of catheter wear-time. Both catheter types produced high intra- and intersubject day-to-day insulin absorption variability. The IC caused significantly more tissue disruption and lead to irregular changes in tissue morphology. Both catheter types were surrounded by a layer of inflammatory tissue that varied in composition, thickness, and density over time. A catheter that was manually inserted by pushing a sharp tip through the skin caused more trauma and variability than a 90° Teflon cannula with automated insertion.

***Conclusions:*** Insulin absorption variability could be attributed to the layer of inflammatory tissue, which may function as a mechanical barrier to insulin flow into adjacent vascular tissue. The impact of the acute inflammatory tissue response on insulin absorption has to be considered in future catheter designs. A catheter that was manually inserted by pushing a sharp tip through the skin caused more trauma and variability than a 90° Teflon cannula with automated insertion.

## Introduction

Worldwide, ∼1 million people manage their diabetes with an insulin pump, rapid acting insulin, and a continuous subcutaneous insulin infusion (CSII) catheter.^1^ Insulin is continuously delivered through the CSII catheter at a variable basal rate between meals and during sleep. Diabetic patients deliver a bolus of insulin according to meal size, composition, insulin to carbohydrate ratio, preprandial glucose concentration, and an estimate of residual insulin-on-board.^1–4^ Despite recent improvements in CSII catheter design and method of insertion, the CSII catheter remains the weak link of an insulin infusion system for the management of type 1 diabetes.^5–7^ Patients are instructed to insert a new CSII set every 2 to 3 days because insulin absorption into the circulation becomes more variable and less reliable over time.^6,8,9^ Catheters are routinely replaced when a bolus dose of insulin fails to produce the expected glucose lowering effect, defined as unexplained hyperglycemia.

The mechanisms that cause variable and unreliable insulin absorption from a CSII catheter into the circulation are poorly understood.^10–13^ When a CSII catheter is inserted, it causes an acute inflammatory response of the adipose tissue.^14^ A layer of inflammatory tissue forms around each CSII cannula that contains plasma proteins, platelets, red blood cells, white blood cells, and cellular debris.^14–16^ The degree of inflammation is dependent on catheter material, shape, and wear-time and may influence insulin absorption.^14,17^

The success of insulin pump therapy may further be dependent on the robustness of insertion methods to avoid issues such as catheter kinking and therefore failed delivery of insulin into the tissue.^18,19^ We hypothesize that the flow of insulin from a CSII catheter into adjacent vascular tissue is affected by the changing composition and architecture of the inflammatory tissue layer surrounding the cannula, causing insulin absorption pharmacokinetics (PK) to be variable. We believe that this variability is further influenced by the catheter material, shape, and insertion method.

The goal of this pilot study was to evaluate the PK of lispro insulin absorption and the tissue response to two different CSII catheter materials (Teflon and a flexible metal-ceramic alloy) and designs implanted in the subcutaneous tissue of six swine for 5 days.

## Research Design and Methods

We performed an observational study in six swine to evaluate the PK of insulin lispro (U-100 Humalog; Eli Lilly) infused through CSII catheters implanted for 5 days. Six commercial CSII catheters (CCs) and six investigational CSII catheters (ICs) were inserted into the subcutaneous tissue of each swine. Lispro insulin and saline were infused through the CCs and ICs for 5 days using battery powered insulin pumps and the same basal/bolus pattern of delivery. Additional CCs and ICs were filled with saline and capped (Supplementary Table S1; Supplementary Data are available online at www.liebertpub.com/dia). Insulin PK (*C*_max_, *t*_max_, AUC, AUC60) following a 5-U bolus were evaluated on days 1, 3, and 5. The tissue surrounding the 72 CSII was excised and pathologically analyzed to elucidate the relationship between the PK of insulin absorption and the inflammatory tissue response to trauma and the CSII cannula.

### In vivo swine study (*n* = 6)

The study was performed in six nondiabetic ambulatory female swine (68.5 ± 3.6 kg). The protocol was approved by the Institutional Animal Care & Use Committee of Thomas Jefferson University. General anesthesia and aseptic surgical technique were used to implant central venous catheters (CVC, CP2 vascular access ports; Access Technologies, IL) into the left innominate vein (for blood sample acquisition) and the distal superior vena cava (for glucose infusion). The CVC ports were flushed every 1–3 days using a Huber needle and low-dose heparin solution. One week later, each swine was given general anesthesia and six CCs (Inset™; UnoMedical, Denmark) and six ICs were inserted into the subcutaneous tissue of the abdomen using an aseptic technique according to the manufacturer's instructions. In total, 72 CSII were implanted in six swine.

The CC had a stable plastic platform with an adhesive base for attachment to the skin. The 6 mm Teflon cannula was inserted at a 90° angle through the skin and subcutaneous tissue over a sharp 27-gauge steel needle. Insertion force was applied to the catheter using a spring-loaded introducer. The needle was removed leaving the Teflon cannula within the subcutaneous tissue throughout the 5-day study (Supplementary Fig. S1, left). The IC had a stable plastic platform with an adhesive base for attachment to the skin. The platform was an open clam-shell design with a side hinge. Closing the platform manually guided the curved cannula through the skin and into the subcutaneous tissue at an insertion angle of ∼60°. The needle was thin and slightly flexible with a sharp tip (Supplementary Fig. S2, right). The IC cannula remained within the subcutaneous tissue throughout the 5-day study. CSII catheters were carefully inspected to confirm correct insertion through the skin and into the subcutaneous tissue.

Two CCs and two ICs were connected to insulin pumps (OneTouch Ping; Animas Corporation, West Chester, PA) containing rapid acting lispro insulin. Two CCs and two ICs were connected to insulin pumps containing saline solution. The remaining two CCs and two ICs were primed with saline and did not receive an infusion (Supplementary Table S1). CSII were secured with Tegaderm™ adhesive bandages and the eight insulin pumps were secured within the pockets of a custom vest (Lomir Biomedical, Inc., Supplementary Fig. S2). Insulin lispro was continuously infused through the four CSII catheters at a low basal rate (0.2 U/h/catheter = 0.8 U/h). Insulin and saline were infused using the same basal/bolus pattern throughout the 5-day study.

The subcutaneous tissue glucose concentration was monitored using commercial glucose sensors (SEVEN PLUS; Dexcom, Inc.) calibrated using CVC blood samples and a reference glucose analyzer (YSI-2300STAT Plus™; Yellow Springs Instruments). A battery powered pump (Ipump Pain Management System, Baxter MN) continuously infused a solution of 50% dextrose into a CVC between PK studies to minimize the risk for hypoglycemia.

### Insulin PK

PK studies were performed on days 1, 3, and 5 after CSII insertion. On day 1, animals recovered from general anesthesia for ∼2 h before starting the PK study. Blood was sampled from a CVC every 10 min for ∼2 h, followed by every 15 min for 1 h before administering the second bolus and repeating the sampling schedule. When blood sample acquisition from a CVC was not possible, samples were obtained from the alternate CVC or occasionally omitted. The swine were able to stand up, lie down, and consume food (apples, biscuits) and water during the PK study. The concentration of blood glucose (BG) was measured in duplicate using the YSI analyzer. Samples were centrifuged to plasma and stored at −80°C for subsequent insulin assay. A stable baseline was established for ∼30 min (three to five BG values).

We then infused a 5-U bolus of insulin into a CC or an IC in a randomized order. The concentration of BG was maintained between 100 and 160 mg/dL by adjusting an intravenous infusion of 20% dextrose every 5–10 min for 3 h. A second baseline was then established for ∼30 min to ensure that BG levels have returned to baseline values after the morning insulin bolus, followed by a 5-U bolus of insulin infused into the alternate CSII catheter. The afternoon PK study ended ∼3 h later. Thus, two PK studies were performed on days 1, 3, and 5 in the same swine using the same CC and IC each day.

The concentration of lispro insulin was measured in plasma samples by PreClinOmics, Inc. (Indianapolis, IN) using a porcine insulin ELISA and an Iso-insulin ELISA (Mercodia AB, Uppsala, Sweden). The lispro insulin concentration was calculated by subtracting the porcine insulin from the total insulin concentration. A baseline was calculated for each PK study averaging the last three to five values before the bolus was administered (*t* = 0), and subtracting this value from insulin concentrations at each time point. Maximal plasma insulin levels (*C*_max_) and time-to-peak (*t*_max_) were determined.

The areas under the curve (AUC) of insulin concentration (baseline subtracted) versus time were calculated between the time of bolus and 60 min (AUC60) and 160 min (AUC) after the bolus. We applied the linear trapezoidal method using the PKSolver add-in for Microsoft Excel.^20^ For a given time interval (*t*_2_−*t*_1_), the integral was calculated by ½·(*C*_1_ + *C*_2_)·(*t*_2_−*t*_1_). AUC equals the sum of the integrals. The inter- and intrasubject coefficient of variation (CV) was calculated for AUC, AUC60, *C*_max_, and *t*_max_ for each day and across all study days.

### Tissue histology

The swine were euthanized after completion of the third PK study on day 5. A 50 μL bolus of methylene blue dye was then infused into the CCs and ICs to determine whether dye migrated onto the skin surface. The skin and subcutaneous tissue surrounding the 72 catheters were excised and fixed in 10% formalin for 3 days. The tissue was grossed and processed using a long dehydration protocol for fatty tissue.^21^ Specimens were sectioned perpendicular or parallel to the skin surface depending on the location and angle of the cannula in the excised tissue specimen. Four-micrometer-thick tissue sections were stained with hematoxylin and eosin (nuclei are stained blue/purple, extracellular matrix [ECM] and cytoplasm are stained in pink), Masson's trichrome (collagen/connective tissue is stained blue, muscle fibers and erythrocytes are stained bright red, and cytoplasm is stained pink), and a reticulin stain (type III collagen is stained silver).

A surgical pathologist analyzed the stained tissue for no, mild, moderate, or severe (grades 0, 1, 2, or 3, respectively) hemorrhage, reticular fiber disruption, fibrin deposition, fat necrosis, and collagen deposition compared with unaffected tissue. This grading system was newly established by the lead pathologist of this study who predefined these criteria before analyzing all samples. The pathologist remained blinded throughout the study. The size of the debris field was measured (mm) using the Aperio ScanScope image capture device and ImageScope software (Leica Biosystems). Topography of tissue planes was considered regular if subcutaneous tissue was pushed down evenly in the direction of the cannula tip and irregular or intermediate otherwise. Irregular or regular reservoir geometry described the shape of the debris field surrounding the catheter tip.

Fisher's exact test was used to evaluate the differences in histology profiles of CCs and ICs. A chi-squared test was applied to analyze differences in methylene blue dye leakage.

## Results

### Tissue histology surrounding a CSII catheter

**FIG. 1.**
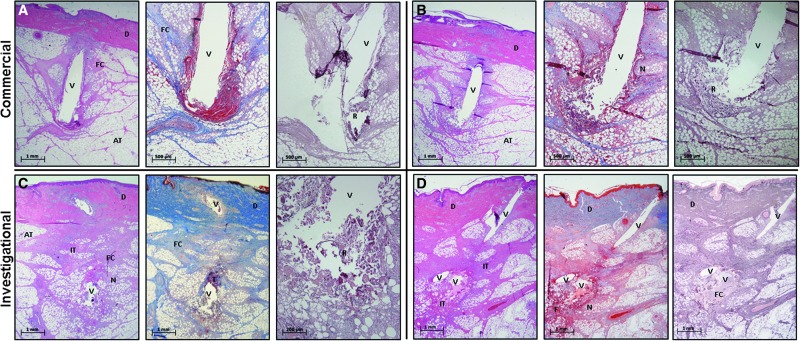
Examples of subcutaneous AT histology surrounding two CCs **(A, B)** and two ICs **(C, D)** stained (from left to right) with hematoxylin and eosin, Masson's trichrome, and reticulin III. CSII cannulas were removed after tissue fixation leaving a void (V) that previously contained damaged adipose cells, capillaries, lymphatics, connective tissues, extracellular matrix, and thrombus (T). Subcutaneous tissue was pulled downward by cannula insertion leading to regions of compressed connective tissue and adipose cells. Dense areas of dark red cells were produced by hemorrhage, thrombosis, and fibrin deposition (F). Neutrophils, macrophages, fibroblasts, and other immune cells migrated into the damaged tissue to form regions of acute IT that nearly surrounded the implanted cannula. This layer of inflammatory tissue varied in thickness, composition, and continuity at various locations along the cannula shaft. Damaged cells and connective tissue were more pronounced at the cannula's distal end. Reticular fibers (R) were disrupted near the cannula tip. Areas of adipose cell necrosis (N) would have contained free fatty acid, triglyceride, cytoplasm, and organelles. Fibroblasts deposited FC throughout the wound (light pink or light blue). This layer of inflammatory cells, FC, and fibrin nearly surrounded the insertion channel (V) of each specimen. Dermal cells (D) commonly migrated downward around the cannula shaft. The ICs produced two voids in the tissue specimens due to the curved shape of the cannula. The CCs produced a single void due to the straight 6 mm Teflon cannula. AT, adipose tissue; CCs, commercial CSII catheters; CSII, continuous subcutaneous insulin infusion; FC, fresh collagen; ICs, investigational CSII catheter; IT, inflammatory tissue.

In general, the tissue surrounding the CSII cannula on day 5 of implantation resembled a clean surgical wound with a foreign body, rather than normal dermis and subcutaneous tissue (Supplementary Fig. S3). Cells, capillaries, lymph vessels, connective tissues (collagen, elastin, reticular fibers), and ECM were damaged along the path of cannula insertion. Thrombus formed around many of the cannulas due to damaged arterioles, capillaries, and venules (Fig. 1A center). All of the specimens had regions of adipose cell necrosis (Fig. 1A–D) and acute inflammation. Although not visualized, lysed adipose cells would have released their contents (cytoplasm, nucleus, organelles, and triglycerides) into the wound.

Neutrophils, macrophages, fibroblasts, and lymphocytes migrated into the areas of tissue damage from the local capillaries (dense purple and pink areas within subcutaneous tissue in hematoxylin and eosin sections, Fig. 1A–D, Supplementary Figs. S4–S15). Thrombus and immune cells produced a layer of fibrin and acute inflammatory tissue that partially or completely surrounded the implanted cannula (Fig. 1A, B, center, Supplementary Figs. S6–S17). Fibroblasts produced a variable amount of fresh collagen (stained light blue in the trichrome stains compared to the darker blue of mature collagen) that added thickness and density to this surrounding layer of inflammatory tissue (Fig. 1A–C, center, Supplementary Figs. S6–S17).

Several specimens showed a downward migration of epidermis/dermis into the insertion channel (Supplementary Figs. S8–S10). Tables 1 and 2 summarize the surgical pathologist's description of the tissue response to CSII implantation for 5 days (*n* = 72; 36 CCs and 36 ICs). The CCs elicited significantly less reticular fiber disruption, fat necrosis, hemorrhage at the cannula tip, a regular tissue topography, and a smaller debris field compared with the ICs. The ICs produced a larger debris field with irregular topography of tissue planes and irregular reservoir geometry (*P* < 0.001). The layer of inflammatory tissue surrounding the CC and IC cannula varied in thickness, density, composition, and continuity.

**Table 1.** Tissue Histology Data

	*CSII catheter*	*None*	*Mild*	*Moderate*	*Severe*	
Reticular fiber disruption	CC	1 (2.8%)	29 (80.6%)	6 (16.7%)	0	^***^
	IC	0	6 (16.7%)	9 (25.0%)	21 (58.3%)	
Fibrin deposition in reservoir	CC	1 (2.8%)	10 (27.8%)	15 (41.7%)	10 (27.8%)	
	IC	0	8 (22.2%)	21 (58.3%)	7 (19.4%)	
Collagen deposition at tip	CC	4 (11.1%)	26 (72.2%)	6 (16.7%)	0	
	IC	1 (2.8%)	23 (63.9%)	11 (30.6%)	1 (2.8%)	
Fat necrosis	CC	1 (2.8%)	22 (61.1%)	13 (36.1%)	0	^***^
	IC	0	7 (19.4%)	14 (38.9%)	15 (41.7%)	
Hemorrhage at tip base	CC	1 (2.8%)	27 (75.0%)	8 (22.2%)	0	^*^
	IC	1 (2.8%)	17 (47.2%)	17 (47.2%)	1 (2.8%)	

*Changes in tissue morphology*		*Regular*	*Intermediate*	*Irregular*		
Reservoir geometry	CC	29 (82.9%)	—	6 (17.1%)		^***^
	IC	11 (30.6%)	—	25 (69.4%)		
Topography of tissue planes along insertion^a^	CC	30 (85.7%)	5 (14.3%)	0		^***^
	IC	1 (2.8%)	19 (52.8%)	16 (44.4%)		

		*Depth (mm) ± SD*				
Debris field (mean)	CC	3.2 ± 1.1				^***^
	IC	4.9 ± 1.4				

Values in brackets are a percent of 36 CCs and 36 ICs total.

^a^ Tissue morphology for Swine1-CC1 was not evaluated due to incomplete/superficial insertion of catheter; ^***^P < 0.001. ^*^P < 0.05 significant difference between catheter types.

CC, commercial CSII catheter; CSII, continuous subcutaneous insulin infusion; IC, investigational CSII catheter.

**Table 2.** Tissue Histology Data According to Type of Infusion (Insulin, Saline, None)

	*Infusion*	*None*	*Mild*	*Moderate*	*Severe*	
Reticular fiber disruption	Insulin	1 (4.2)	9 (37.5)	6 (25.0)	8 (33.3)	
	Saline	0	13 (54.2)	3 (12.5)	8 (33.3)	
	None	0	13 (54.2)	6 (25.0)	5 (20.8)	
Fibrin deposition in reservoir	Insulin	1 (4.2)	2 (8.3)	11 (45.8)	10 (41.7)	^*^
	Saline	0	9 (37.5)	11 (45.8)	4 (16.7)	
	None	0	7 (29.7)	14 (58.3)	3 (12.5)	
Collagen deposition at tip	Insulin	2 (8.3)	16 (66.7)	5 (20.8)	1 (4.2)	
	Saline	2 (8.3)	16 (66.7)	6 (25.0)	0	
	None	1 (4.2)	17 (70.8)	6 (25.0)	0	
Fat necrosis	Insulin	1 (4.2)	5 (20.8)	12 (50.0)	6 (25.0)	
	Saline	0	14 (58.3)	5 (20.8)	5 (20.8)	
	None	0	10 (41.7)	10 (41.7)	4 (16.7)	
Hemorrhage at tip base	Insulin	1 (4.2)	13 (54.2)	10 (41.7)	0	
	Saline	1 (4.2)	15 (62.5)	7 (29.2)	1 (4.2)	
	None	0	16 (66.7)	8 (33.3)	0	

Values in brackets are a percent of 36 CCs and 36 ICs total; ^*^P < 0.05 significant difference between infusion types.

There was no statistically significant difference in fibrin or collagen deposition at the cannula tip. The tissue histopathology surrounding the CSII infused with lispro insulin had a greater amount of fibrin in the reservoir, but was generally similar to the histology surrounding catheters infused with saline or not infused (Table 2 and Supplementary Table S2).

### Insulin PK

Table 3 summarizes the PK data for the CCs and ICs infused with a 5-U insulin bolus. CV for intersubject (between) and intrasubject (within) variability are summarized in Table 4 and Supplementary Table S3. The mean AUC and *C*_max_ were higher and *t*_max_ shorter for insulin administered through the CCs compared to the ICs. However, these data could not be analyzed for statistical significance due to the small number of studies. Data for swine 1 were excluded before PK analysis, due to faulty insertion (kinking) causing inadequate penetration into the subcutaneous tissue, leading to limited insulin absorption. We furthermore could not calculate the AUC for the CC in swine 6, as we were unable to collect blood samples 20 min after bolus administration for a period of 1.5 h in this animal.

**Table 3.** Mean Values for AUC, AUC60, *C*_max_, and *t*_max_ for Both Types of CSII Catheters on Days 1, 3, and 5

*Catheter type*	*Day*	${ \rm { AUC } } \left( { { \frac { { \rm { mU } } } { \rm { L } } } { \rm { h } } } \right)$	${ \rm { AUC60 } } \left( { { \frac { { \rm { mU } } } { \rm { L } } } { \rm { h } } } \right)$	${ { \rm { C } } _ { \max } } \left( { { \frac { { \rm { mU } } } { \rm { L } } } } \right)$	${{ \rm{t}}_{ \max }} \left( { \min } \right)$
CC (*n* = 5^a^ swine)	1	82.7 ± 37.6	50.9 ± 12.9	90.7 ± 26.8	35.0 ± 17.3
	3	63.1 ± 22.8	47.0 ± 25.6	73.9 ± 41.0	20.0 ± 12.2
	5	71.1 ± 29.4	54.9 ± 20.8	114.7 ± 30.1	12.0 ± 4.5
IC (*n* = 6 swine)	1	68.5 ± 36.0	44.3 ± 23.2	120.0 ± 59.3	43.3 ± 28.8
	3	63.1 ± 51.8	42.7 ± 40.0	115.0 ± 86.2	20.0 ± 6.3
	5	40.1 ± 37.6	29.0 ± 31.3	105.1 ± 87.7	33.3 ± 18.6

Data are provided as mean ± SD.

^a^ PK from swine 1 excluded due to incomplete insertion of CC1 catheter.

AUC, areas under the curve; PK, pharmacokinetics.

**Table 4.** Coefficients of Variation for Inter- and Intrasubject Variability for Each Day and Over All Days

*CV type*	*Catheter*	*Day*	*CV AUC, %*	*CV AUC60, %*	*CV* C_*max*_*, %*	*CV* t_*max*_*, %*
Between animals	CC	1	45.6	25.3	29.5	49.5
	CC	3	36.1	54.5	55.6	61.2
	CC	5	41.3	38.0	26.2	37.3
Between animals	IC	1	52.5	52.5	49.4	66.4
	IC	3	82.1	93.7	75.0	31.6
	IC	5	93.8	107.9	83.4	55.9
Between animals	CC	All	13.7	7.8	22.0	52.3
	IC	All	26.4	21.7	6.7	36.3
Within animals	CC	All	30.8	25.5	37.0	39.2
	IC	All	69.6	83.8	49.5	55.5

CV, coefficient of variation.

**FIG. 2.**
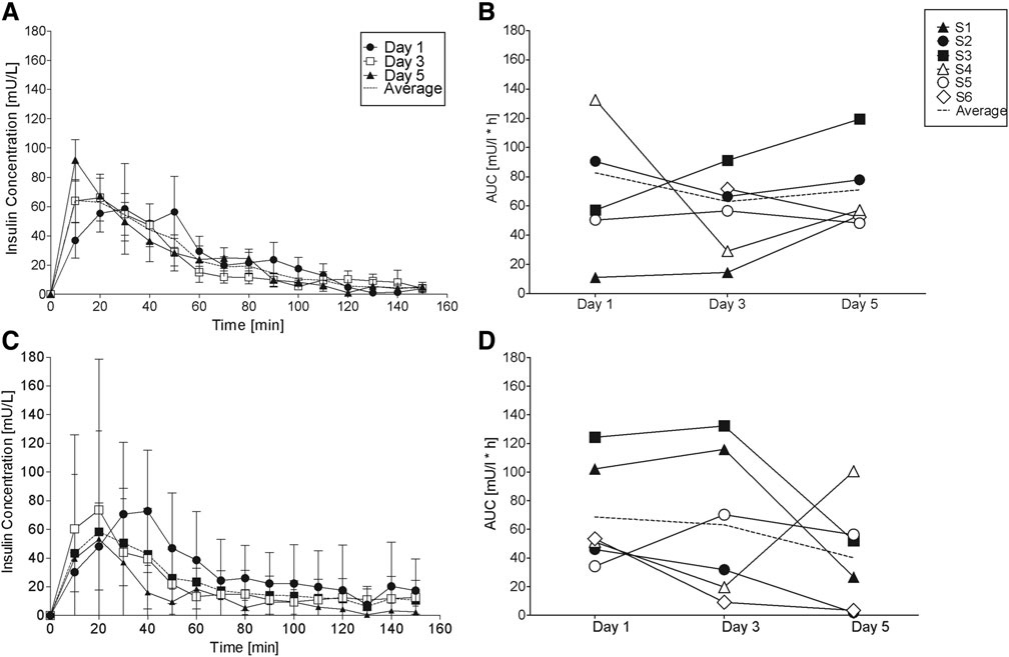
Insulin concentration versus time curves **(A, C)** and area under the insulin absorption curve **(**AUC, **B, D)** for CC (commercial) and IC (investigational) CSII catheters. **(A)** CC and **(C)** IC show average plasma insulin concentration curves on days 1, 3, and 5 of catheter wear-time. Dotted lines represent the average ± SD for the three PK studies. **(B)** CC and **(D)** IC show area under the insulin absorption curve (AUC) over 2.5 h. Dotted line represents the average AUC values for six swine on days 1, 3, and 5 (data from swine 6 on day 1 were excluded because we were unable to obtain blood samples). AUC, areas under the curve; PK, pharmacokinetics.

**FIG. 3.**
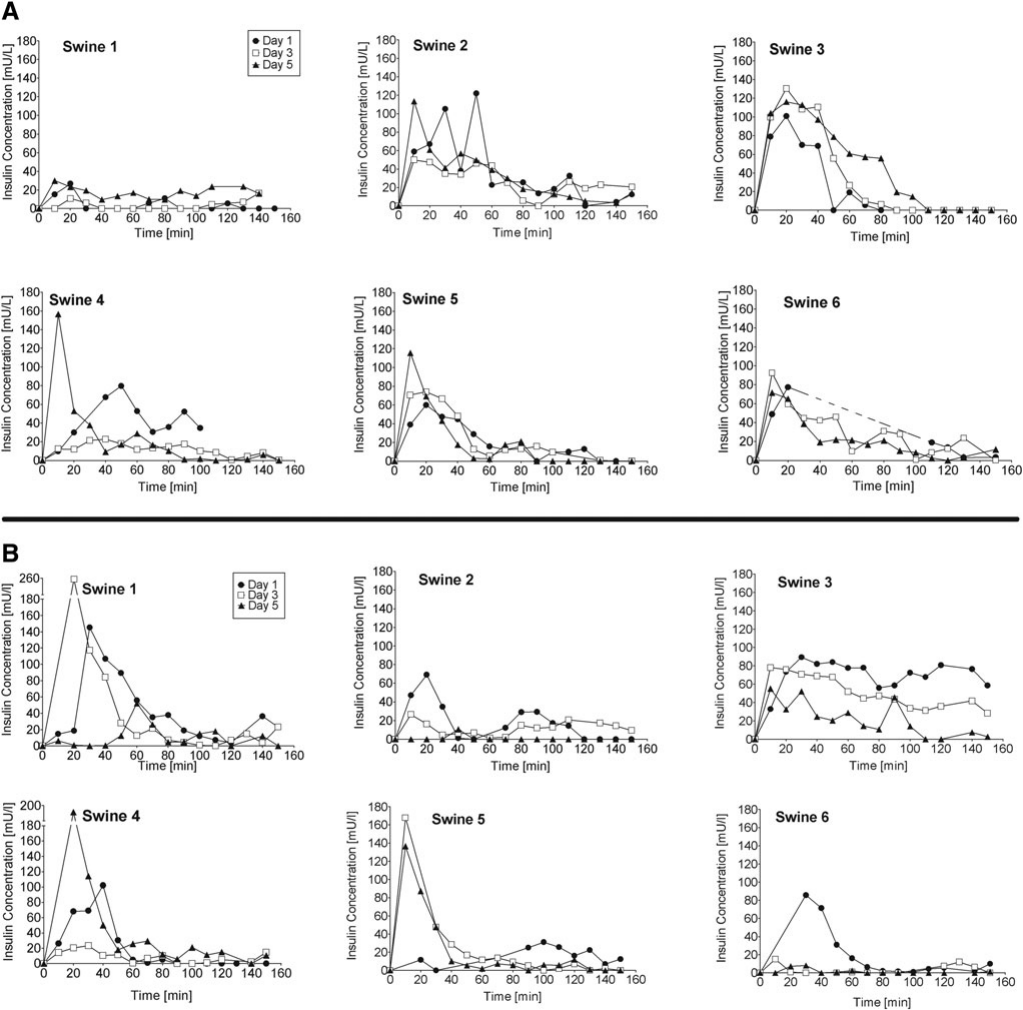
Individual plasma insulin concentration curves for six swine on days 1, 3, and 5 starting at the time of a 5 U insulin bolus infusion (*t* = 0) using **(A)** CCs and **(B)** ICs. (See Supplementary Figs. S16 and S17 for corresponding CSII catheter photos and tissue histology photos.)

The averaged PK data for days 1, 3, and 5 are shown in Figure 2 and the individual PK curves are shown in Figure 3. All of the CCs and ICs produced a high intrasubject and intersubject day to day PK variability (Table 4). The other five CCs produced the fastest insulin absorption (*t*_max_ = 12.0 ± 4.5 min), the highest plasma insulin concentration (*C*_max_ = 114.7 ± 30.1 mU/L), and the highest AUC60 (54.9 ± 20.8 mU/L·h) on day 5 of implantation. Insulin absorption from the CCs and was least variable on day 5 and most variable on day 1 (Fig. 2 and Table 4). Two CCs produced low and delayed insulin absorption on day 1, followed by higher insulin absorption on subsequent days. The six ICs produced the highest AUC60, AUC, and *C*_max_ on day 1, the fastest insulin absorption on day 3, and the lowest AUC60, AUC, and *C*_max_ on day 5. Five of the six ICs produced a lower AUC on day 5 compared with day 3 (Table 3). Insulin absorption from the ICs was least variable (range 34–124 mU/L*h, intersubject CV = 52.5%) on day 1 and most variable on day 3 (range 9–132 mU/L*h, intersubject CV = 93.8%). We were unable to detect a statistical relationship between the observed histology results and insulin absorption PK due to the small sample size and high variability. Supplementary Table S4 gives an overview over the histology data and corresponding PK values.

### Insulin pump occlusion alarms, dye leakage, and insulin PK

The CC of swine 1 used for the PK study did not insert fully into the subcutaneous tissue, kinked, leaked a large amount of dye onto the skin surface, and produced very limited insulin absorption into the circulation (Fig. 2B). No other CC leaked dye onto the skin surface (35/36). One of the CCs leaked a large amount of dye into the hub (3%), five CCs leaked a small amount into the hub (14%), and four CCs had a pump occlusion alarm (11%). Dye leakage and occlusion alarms occurred in CCs that were infused with insulin lispro, saline, and not infused (Supplementary Table S5). The CC of swine 2 used for the PK study produced a pump occlusion alarm on day 3 only. No other CC used for the PK study developed an occlusion alarm. The ICs of swine 1, 2, 3, and 4 used for the PK study produced one or more occlusion alarms. The IC of swine 1, 2, and 6 leaked dye into the CSII hub and produced limited insulin absorption on days 3 and/or 5 (Fig. 2D).

## Discussion

To our knowledge, this is the first study comparing insulin PK and tissue histopathology surrounding two different types of CSII catheters in the same swine while infusing insulin, saline, or no infusion. The commercial and ICs had distinct designs, materials, and methods of insertion that produced a significant difference in the inflammatory tissue response and distinct patterns of insulin absorption. A surgical pathologist analyzed the tissue specimens using an objective grading system with each swine acting as its own control. The study was limited by the small sample size and the difficulty in controlling variables that may affect insulin absorption into the subcutaneous vascular tissue in awake swine, such as temperature, activity level, and food intake. Many CCs and ICs produced one or more pump occlusion alarms, leaked dye/insulin into the hub or onto the skin surface. In addition, histology did not always provide a complete picture of the tissue surrounding the CSII cannula, as localization of the full insertion channel was sometimes difficult. At the time of plasma insulin measurement, no lispro-specific ELISA was commercially available and insulin concentrations were calculated indirectly by subtracting porcine insulin from total insulin content. Unfortunately, this indirect calculation adds to the calculated intra- and intersubject variability.

An ideal CSII catheter would produce fast and consistent insulin PK for an extended period of time (4–14 days) with no occlusions, leaks, or infections. Patients are currently educated to replace their CCs every 2 to 3 days to minimize the risk of infection and because insulin absorption from the subcutaneous tissue into the circulation becomes more variable and less reliable over time. Patients commonly experience an insulin bolus that fails to produce the expected glucose lowering effect, leading to persistent hyperglycemia.^6,19^ Insulin absorption (PK) in this swine study was variable when insulin was infused through either type of CSII catheter on days 1, 3, and 5. This observation is consistent with published animal studies, human clinical trials, and routine clinical care.^5,6,22,23^ Of interest, the CCs produced the fastest insulin absorption, the highest plasma insulin concentration, and the greatest AUC60 on day 5 of implantation. Insulin absorption from the CCs was least variable on day 5 and most variable on days 1 and 3. The day 5 observations are of interest, as current recommendations do not recommend the patient to wear a CSII set until day 5, when insulin absorption may recover. Studies have shown that some patients indeed successfully wear CSII catheters up to 7 days.^19,24^ In contrast, the ICs produced the highest AUC, AUC60, and *C*_max_ on day 1, the fastest *t*_max_ on day 3, and the lowest AUC, AUC60, and *C*_max_ on day 5. Figures 1 and 2 illustrate the importance of reviewing the individual PK curves from each experiment to acknowledge the large dose-to-dose and day-to-day variability. The individual PK curves are highly variable in contrast with the average PK curves. Ten of the 36 PK studies resulted in a limited and/or delayed insulin absorption curve. The three ICs that produced low insulin absorption on day 5 had a pump occlusion alarm and/or leaked dye into the hub. The decreased uptake of insulin when administered through the IC may primarily be explained by issues in the connector between catheter and tubing. Methylene blue dye often accumulated in the hub indicating a technical obstruction of insulin flow.

The tissue response around CCs was characterized by a central area of adipose tissue debris surrounded by a layer of thrombus infiltrated with immune cells. Many of the specimens had a continuous or near-continuous layer of inflammatory tissue surrounding the CSII cannula, while other specimens had a discontinuous layer with normal adipose tissue adjacent to the CSII cannula. Compared with the tissue response to IC implantation, the tissue layer surrounding the Teflon cannula had more regular connective tissue topography, more regular reservoir geometry, a smaller debris field, less reticular fiber disruption, less fat necrosis, and less hemorrhage within the reservoir. The shape of the IC possibly contributed substantially to the extent of trauma due to its sharp tip, which may have continuously disrupted tissue in the vicinity. Round, soft shapes are known to be better tolerated that sharp edges.^17,25^ In general, the ICs produced a similar, yet more pronounced, tissue response characterized by a central core of adipose tissue debris surrounded by a layer of fibrin thrombus infiltrated with inflammatory cells. We did not observe a difference in tissue response between infusion types, except for increased fibrin when insulin was infused.

Although the inflammatory response may be different from humans, the swine has been described as the most adequate model for human wound healing.^26–29^ Like humans it has a thick epidermis (70–150 μm), sparse body hair, and a similar composition of the skin lipid layer.^26,29^ The subcutaneous adipose tissue of the swine has a similarly high elastic and collagen fiber content and the animals show a healing mechanism comparable to humans.^26,27,29^

When insulin is infused, an insulin pump produces a hydrostatic pressure differential between the inside of the CSII cannula and the adjacent subcutaneous tissue, causing insulin to flow into the tissue along the path of least resistance. Slow increases and abrupt decreases in insulin pump back pressure suggest that a bolus dose of insulin may accumulate within the layer of inflammatory tissue, distend the tissue, and then abruptly travel into adjacent tissue through a pathway with lower resistance.^30^ Insulin may flow into the connective tissue septa that surround the adipose cells, remain within the surrounding layer of inflammatory tissue, and/or flow upward along the cannula shaft onto the skin surface.^30,31^ We hypothesize that a layer of inflammatory tissue that is thick, dense, and/or continuous may prevent or delay insulin from reaching the adjacent vascular tissue. Insulin may travel through one or more holes and defects in the layer of inflammatory tissue when the hydrostatic pressure exceeds the resistance to flow. Results from this study indicate faster and better absorption of insulin on day 5, however, with a fairly high interindividual CV of 41% for the AUC of the commercial and 94% for the investigational catheter. The intrapatient CV of 37% for *C*_max_ and 39% for *t*_max_ are comparable with recently published human data.^32^ This high variability may be explained by the inconsistency of the mechanical inflammatory layer surrounding the cannula of a CSII catheter. Furthermore, the acute inflammatory response and the infusion of insulin may directly increase local blood flow,^33–38^ which may contribute to increased insulin uptake on day 5 of catheter wear-time.

Numerous factors can affect the efficiency and time course of insulin absorption into the circulation. One of them being failed insertion of the CSII catheter. The clam-shell mechanism of the IC was sometimes difficult to handle and the insertion angle of the cannula was not consistent among samples. One CC kinked below the skin, leading to inadequate insulin delivery. Both manual and supported insertions are prone to insertion failure and the optimal insertion angle is not always achieved. Ideally, the insertion of a catheter is swift and easy and the insertion angle reproducible. Insulin absorption can further be negatively influenced by insulin traveling upward onto the skin due to an obstruction of insulin flow into the adjacent tissue. We saw that issues with the catheter hub can lead to physical obstructions of insulin flow into the catheter and tissue. When unnoticed by the patient, blood glucose control may become challenging.

In conclusion, we found substantial differences in the inflammatory response to two different catheter shapes and materials and an increase in intra- and interindividual variability of insulin absorption over catheter wear-time. Maximum insulin plasma concentration levels did not change or increased with wear-time. A catheter that was manually inserted by pushing a sharp tip through the skin caused more trauma and variability than a 90° Teflon cannula with automated insertion.

Additional animal and human studies are needed to better understand the effects of initial tissue damage and ongoing tissue damage on the composition, thickness, density, and continuity of the layer of inflammatory tissue that surrounds a CSII cannula; and how this immune response affects the rate and precision of insulin absorption over time. The impact of the acute inflammatory tissue response on insulin absorption may have to be considered in future catheter designs.

## Supplementary Material

Supplemental data
