# Supplementary material for: Detailed Analysis of Insulin Absorption Variability and the Tissue Response to Continuous Subcutaneous Insulin Infusion Catheter Implantation in Swine
Source: Diabetes Technol Ther. 2017 Nov 1;19(11):641–50. doi: 10.1089/dia.2017.0175 (PMC5689134; doi:10.1089/dia.2017.0175)
Supplement: Supplemental data [file Supp_Data.docx]

**SUPPLEMENTARY DATA**

Table S1 – CSII Catheter Overview.

| Catheter no. | Infusion | Catheter no. | Infusion |
| --- | --- | --- | --- |
| CC1 | lispro (PK+basal) | IC1 | lispro (PK+basal) |
| CC2 | lispro (PK+basal) | IC2 | lispro (PK+basal) |
| CC3 | Saline | IC3 | Saline |
| CC4 | Saline | IC4 | Saline |
| CC5 | None | IC5 | None |
| CC6 | None | IC6 | None |
| CC: commercial CSII catheter, IC: investigational CSII catheter. The insertion pattern was randomized. Insulin lispro was continuously infused through CC1, CC2, IC1 and IC2 for 5 days at a slow basal rate (0.2 units/hour). A bolus of insulin lispro (1.5 units) was infused through each CSII three times per day. A glucose-clamp PK study was performed using a CC or IC in the morning and afternoon on days 1, 3, and 5 of implantation (randomized order). Preservative free saline was infused through CC3, CC4, IC3, and IC4 for 5 days using the same basal/bolus pattern. CC5, CC6, IC5, and IC6 were filled with saline, capped and not infused. The CSII catheter-tissue specimens were explanted after the PK study on day 5. | | | |


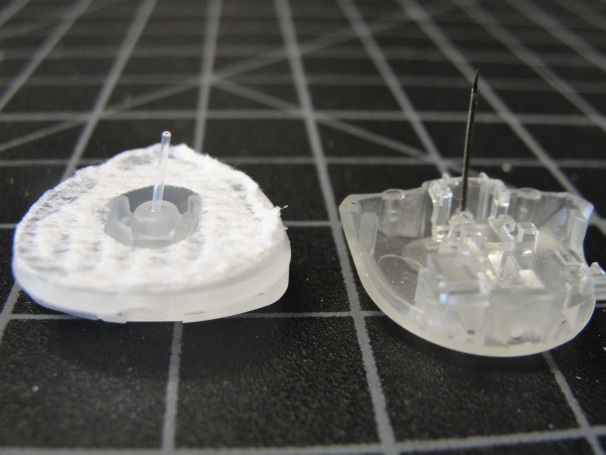


**Figure S1:** Commercial CSII catheter (CC) with 6mm Teflon cannula and insertion needle removed (left) and Investigational CSII catheter with curved cannula containing sharp needle on distal end (right).


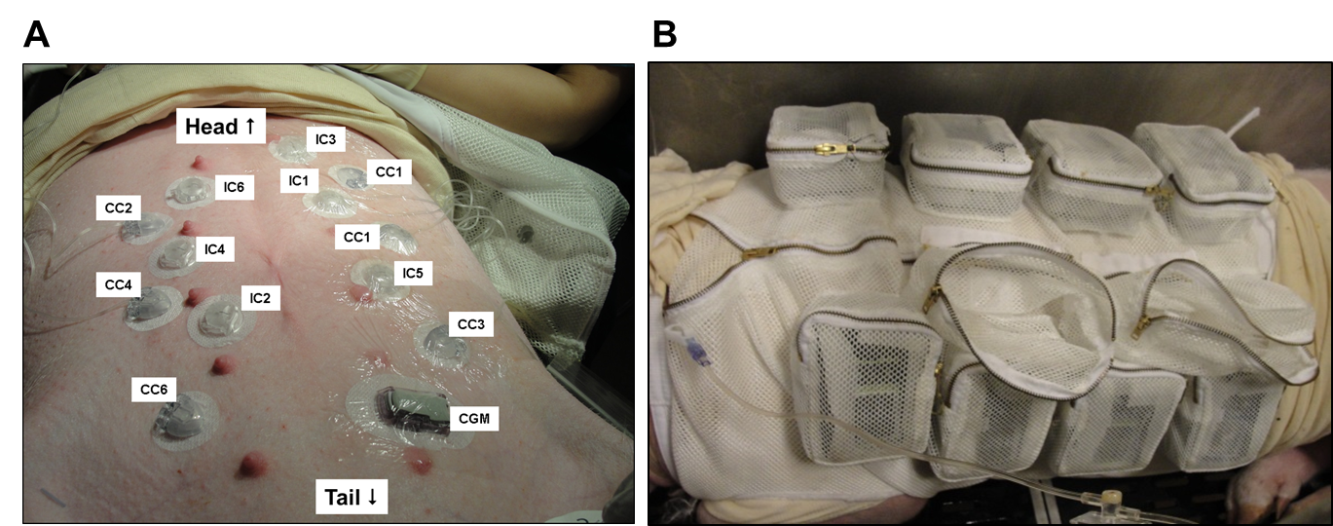


**Figure S2** – ***A:*** 6 CC and 6 IC catheters were inserted into the abdominal subcutaneous tissue of each swine. The CC2 and IC2 catheters in this photo were used for the insulin PK study. The CSII catheters were covered with flexible plastic bandages (Tegaderm®) and an elastic undershirt. Each CSII was attached to a commercial insulin pump (Ping- Animas Corporation) housed within the pocket of a custom vest. Two continuous glucose monitors (CGM) were inserted into the subcutaneous tissue of each swine (Seven- Dexcom Corporation) to monitor the concentration of tissue fluid glucose. ***B:*** Ambulatory swine wearing a vest containing 8 insulin pumps. The top pockets housed a battery powered pump that continuously infused glucose solution into a central venous catheter for 5 days.

**Table S2 –** Tissue histology data according to catheter and infusion type.

|  |  | CSII Catheter | None | Mild | Moderate | Severe | Significance |
| --- | --- | --- | --- | --- | --- | --- | --- |
| Reticular Fiber Disruption | Insulin | CC | 0 | 9 (82%) | 2 (18%) | 0 | ^§^ |
|  |  | IC | 0 | 0 | 4 (33%) | 8 (67%) |  |
|  | Saline | CC | 0 | 11 (92%) | 1 (8%) | 0 | *** |
|  |  | IC | 0 | 2 (17%) | 2 (17%) | 8 (67%) |  |
|  | None | CC | 0 | 9 (75%) | 3 (25%) | 0 | * |
|  |  | IC | 0 | 4 (33%) | 3 (25%) | 5 (42%) |  |
| Fibrin Deposition in Reservoir | Insulin | CC | 0 | 0 | 3 (27%) | 8 (67%) | ^§^ |
|  |  | IC | 0 | 2 (17%) | 8 (73%) | 2 (17%) |  |
|  | Saline | CC | 0 | 6 (50%) | 5 (42%) | 1 (8%) | *ns* |
|  |  | IC | 0 | 3 (25%) | 6 (50%) | 3 (25%) |  |
|  | None | CC | 0 | 4 (33%) | 7 (58%) | 1 (8%) | *ns* |
|  |  | IC | 0 | 3 (25%) | 7 (58%) | 2 (17%) |  |
| Collagen Deposition at Tip | Insulin | CC | 0 | 9 (82%) | 2 (18%) | 0 | *ns* |
|  |  | IC | 1 (8%) | 7 (58%) | 3 (25%) | 1 (8%) |  |
|  | Saline | CC | 2 (17%) | 8 (67%) | 2 (17%) | 0 | *ns* |
|  |  | IC | 0 | 8 (67%) | 4 (33%) | 0 |  |
|  | None | CC | 1 (8%) | 9 (75%) | 2 (17%) | 0 | ^§^ |
|  |  | IC | 0 | 8 (67%) | 4 (33%) | 0 |  |
| Fat Necrosis | Insulin | CC | 0 | 4 (36%) | 7 (64%) | 0 | * |
|  |  | IC | 0 | 1 (8%) | 5 (42%) | 6 (50%) |  |
|  | Saline | CC | 0 | 11 (92%) | 1 (8%) | 0 | * |
|  |  | IC | 0 | 3 (25%) | 4 (33%) | 5 (42%) |  |
|  | None | CC | 0 | 7 (58%) | 5 (42%) | 0 | ^§^ |
|  |  | IC | 0 | 3 (25%) | 5 (42%) | 4 (33%) |  |
| Hemorrhage at Tip Base | Insulin | CC | 0 | 7 (64%) | 4 (36%) | 0 | *ns* |
|  |  | IC | 0 | 6 (50%) | 6 (50%) | 0 |  |
|  | Saline | CC | 0 | 11 (92%) | 1 (8%) | 0 | ^§^ |
|  |  | IC | 1 (8%) | 4 (33%) | 6 (50%) | 1 (8%) |  |
|  | None | CC | 0 | 9 (75%) | 3 (25%) | 0 | *ns* |
|  |  | IC | 0 | 7 (58%) | 5 (42%) | 0 |  |
| Chi-square test applied where possible; ^§^Invalid conditions for Chi-square; Values in brackets are a percent of 12 (11) catheters total; ****p*<0.001, **p*<0.05, significant difference between catheter types | | | | | | | |

**Table S3** – Individual values of intra- (within) animal coefficients of variation.

|  | Animal no. | CV (AUC) | CV (AUC60) | CV (C_max_) | CV (t_max_) |
| --- | --- | --- | --- | --- | --- |
| CC | 2 | 15.3% | 26.1% | 41.5% | 99.0% |
|  | 3 | 34.9% | 25.2% | 12.8% | 0.0% |
|  | 4 | 73.4% | 49.9% | 77.9% | 62.4% |
|  | 5 | 8.4% | 9.4% | 34.7% | 34.6% |
|  | 6 | 22.1% | 17.1% | 18.1% | 0.0% |
|  | **Mean** | **30.8%** | **25.5%** | **37.0%** | **39.2%** |
|  | SD | 25.7% | 15.2% | 25.7% | 42.4% |
| IC | 1 | 59.0% | 81.1% | 57.5% | 56.8% |
|  | 2 | 85.1% | 99.9% | 69.4% | 57.7% |
|  | 3 | 43.0% | 34.2% | 9.3% | 21.7% |
|  | 4 | 71.4% | 70.1% | 44.5% | 33.3% |
|  | 5 | 33.9% | 72.4% | 54.4% | 129.9% |
|  | 6 | 125.4% | 145.2% | 61.8% | 33.3% |
|  | **Mean** | **69.6%** | **83.8%** | **49.5%** | **55.5%** |
|  | SD | 33.0% | 36.9% | 21.3% | 39.2% |

**Table S4 –** Tissue histology data and corresponding PK values (Day 5) for all CSII catheters used for PK study

| Swine | Catheter | Debris Field (mm) | Reservoir Geometry | Reticular fiber disruption | Fibrin deposition in reservoir | Collagen deposition at tip | Topography of tissue planes along insertion | Fat necrosis | Hemorrhage at tip base | $AUC(\frac{mU}{L}h)$ | $C_{max}(\frac{mU}{L})$ | $t_{max}(min)$ |
| --- | --- | --- | --- | --- | --- | --- | --- | --- | --- | --- | --- | --- |
| 2 | CC | 3.1 | regular | 1 | 2 | 1 | regular | 1 | 1 | 83.29 | 113.43 | 10 |
| 3 | CC | 3.3 | regular | 1 | 3 | 1 | regular | 1 | 1 | 119.45 | 116.16 | 20 |
| 4 | CC | 1.9 | regular | 1 | 3 | 1 | regular | 2 | 2 | 57.25 | 156.62 | 10 |
| 5 | CC | 2.2 | regular | 2 | 3 | 1 | regular | 2 | 2 | 48.26 | 115.52 | 10 |
| 6 | CC | 2.6 | irregular | 2 | 3 | 1 | intermediate | 2 | 2 | 54.47 | 71.56 | 10 |
| *Mean±SD* | | *2.6±0.6* |  |  |  |  |  |  |  | *72.54±29* | *114.66±30* | *12.00±4* |
| 1 | IC | 7.9 | irregular | 3 | 2 | 1 | irregular | 3 | 2 | 28.30 | 68.17 | 60 |
| 2 | IC | 3.2 | regular | 2 | 2 | 1 | intermediate | 2 | 1 | 1.77 | 47.10 | 50 |
| 3 | IC | 6.8 | irregular | 3 | 2 | 1 | intermediate | 2 | 1 | 54.83 | 85.06 | 30 |
| 4 | IC | 4.3 | irregular | 3 | 2 | 1 | intermediate | 3 | 2 | 100.49 | 269.32 | 20 |
| 5 | IC | 3.8 | irregular | 2 | 1 | 1 | irregular | 2 | 1 | 57.08 | 131.09 | 10 |
| 6 | IC | 4.1 | irregular | 3 | 3 | 0 | irregular | 3 | 2 | 3.27 | 29.76 | 30 |
| *Mean±SD* | | *5.0±1.9* |  |  |  |  |  |  |  | *40.96±38* | *105.08±88* | *33.33±19* |
| Ordinal scaling for histology: 0-none, 1-mild, 2-moderate, 3-severe. A pump occlusion alarm occurred during the bolus infusion of insulin through the IC* of swine 3, 4 and 5. The pump eventually infused a total of 5 units through each IC over a 5 minute period. | | | | | | | | | | | | |

**Table S5** – Dye leakage into the CSII catheter’s hub, dye leakage onto the skin and insulin pump occlusion alarms for all commercial CSII catheters (CC) and investigational CSII catheters (IC).

| Swine | Catheter | No. | Occlusion Alarm | Leak into hub^+^ | Leak onto skin^§^ |
| --- | --- | --- | --- | --- | --- |
| 1 | CC | 1 | No | None | None |
| 1 | CC* | 2 | No | None | Severe |
| 1 | CC | 3 | No | None | None |
| 1 | CC | 4 | No | None | None |
| 1 | CC | 5 | No | None | None |
| 1 | CC | 6 | No | None | None |
| 2 | CC* | 1 | No | Mild | None |
| 2 | CC | 2 | No | None | None |
| 2 | CC | 3 | No | None | None |
| 2 | CC | 4 | No | None | None |
| 2 | CC | 5 | No | None | None |
| 2 | CC | 6 | No | Severe | None |
| 3 | CC | 1 | Yes | None | None |
| 3 | CC* | 2 | No | None | None |
| 3 | CC | 3 | No | None | None |
| 3 | CC | 4 | No | None | None |
| 3 | CC | 5 | No | None | None |
| 3 | CC | 6 | No | None | None |
| 4 | CC* | 1 | No | None | None |
| 4 | CC | 2 | No | None | None |
| 4 | CC | 3 | No | None | None |
| 4 | CC | 4 | Yes | None | None |
| 4 | CC | 5 | No | None | None |
| 4 | CC | 6 | No | None | None |
| 5 | CC | 1 | No | None | None |
| 5 | CC* | 2 | No | None | None |
| 5 | CC | 3 | No | None | None |
| 5 | CC | 4 | No | None | None |
| 5 | CC | 5 | No | None | None |
| 5 | CC | 6 | No | None | None |
| 6 | CC | 1 | Yes | None | None |
| 6 | CC* | 2 | No | Mild | None |
| 6 | CC | 3 | No | Mild | None |
| 6 | CC | 4 | No | Mild | None |
| 6 | CC | 5 | No | None | None |
| 6 | CC | 6 | Yes | Mild | None |
| 1 | IC* | 1 | Yes | Mild | None |
| 1 | IC | 2 | Yes | None | None |
| 1 | IC | 3 | No | None | Severe |
| 1 | IC | 4 | No | None | None |
| 1 | IC | 5 | Yes | None | None |
| 1 | IC | 6 | Yes | None | None |
| 2 | IC | 1 | Yes | Moderate | None |
| 2 | IC* | 2 | No | None | Moderate |
| 2 | IC | 3 | No | Mild | None |
| 2 | IC | 4 | No | Severe | None |
| 2 | IC | 5 | Yes | None | None |
| 2 | IC | 6 | Yes | None | None |
| 3 | IC* | 1 | Yes | Mild | None |
| 3 | IC | 2 | No | Moderate | None |
| 3 | IC | 3 | Yes | None | None |
| 3 | IC | 4 | No | Mild | None |
| 3 | IC | 5 | No | None | Mild |
| 3 | IC | 6 | Yes | Severe | None |
| 4 | IC | 1 | Yes | None | None |
| 4 | IC* | 2 | No | None | None |
| 4 | IC | 3 | Yes | None | None |
| 4 | IC | 4 | No | Severe | None |
| 4 | IC | 5 | Yes | None | None |
| 4 | IC | 6 | No | None | None |
| 5 | IC* | 1 | No | None | None |
| 5 | IC | 2 | Yes | None | None |
| 5 | IC | 3 | Yes | None | None |
| 5 | IC | 4 | No | Severe | None |
| 5 | IC | 5 | No | None | None |
| 5 | IC | 6 | Yes | None | None |
| 6 | IC* | 1 | No | Moderate | None |
| 6 | IC | 2 | Yes | None | None |
| 6 | IC | 3 | No | Moderate | None |
| 6 | IC | 4 | No | Moderate | None |
| 6 | IC | 5 | Yes | None | None |
| 6 | IC | 6 | Yes | None | None |
| ^+^indicating a problem with tubing and flow of liquid, ^§^indicating a problem with insulin absorption into tissue; *catheters used for PK studies; | | | | | |


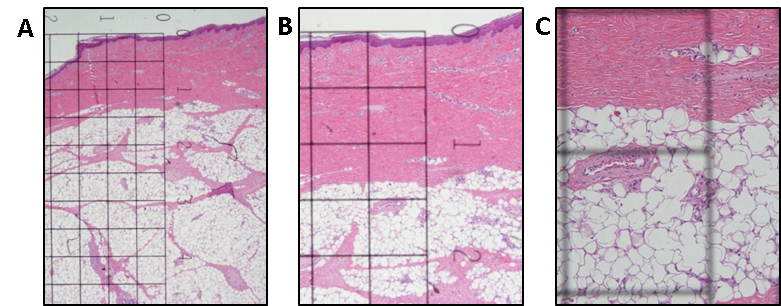


**Figure S3 –** Histology of normal swine tissue showing healthy epidermis, dermis and subcutaneous adipose tissue at ***A:*** low (20x), ***B:*** medium (40x) and ***C:*** high magnification (100x) using H&E stain and light microscopy. Note the thickness, composition, and direction of connective tissue septa that surround adipose lobules. Three or 4 capillary and lymph vessels are typically located within the connective tissue septa that surround around each adipose cell. Larger vessels are located within areas of thicker connective tissue septa. Each grid square = 1 mm².

**Examples of tissue histology and grading by pathologist**

**
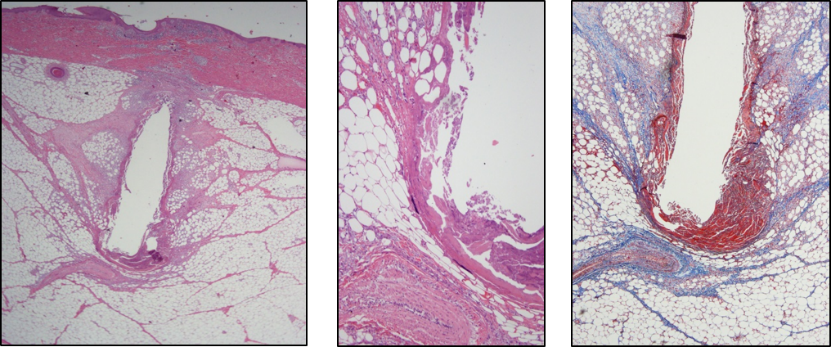
**

**Figure S4** – Swine 5, CC1: Commercial CSII catheter implanted within the subcutaneous tissue of Swine 5 infused with U-100 insulin lispro using a basal/bolus pattern for 5 days. The Teflon cannula was removed from the tissue after fixation- leaving a void in the shape of the cannula. The layer of acute inflammatory tissue varied in thickness, composition and continuity at different locations around the cannula. This layer was composed of compressed connective tissue/adipose cells, thrombus/fibrin, inflammatory cells, and freshly deposited collagen. The pathologists graded the histology: 7.0 mm insertion depth, 2.0 mm debris field, regular reservoir geometry, +3 fibrin deposition in reservoir, +1 reticulum fiber disruption, +1 collagen deposition at tip, + 1 fat necrosis, + 1 hemorrhage at tip, distorted blood vessel integrity at tip base, and regulator typography of tissue planes along path of cannula insertion. From left to right: H&E stain 20x magnification, Trichrome stain 40x magnification, and Reticulum stain 40x magnification. The cannula did not kink during/after insertion, did not produce an insulin pump occlusion alarm, and did not leak blue dye onto the skin on day 5.


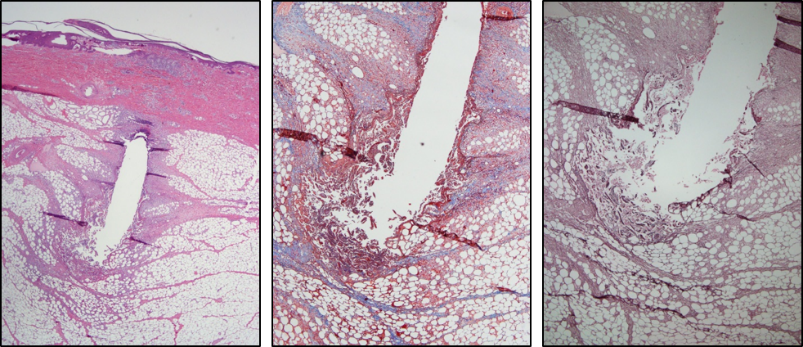


**Figure S5 –** Swine 5, CC2: Commercial CSII catheter implanted within the subcutaneous tissue of Swine 5 infused with U-100 insulin lispro using a basal/bolus pattern for 5 days. The Teflon cannula was removed from the tissue after fixation- leaving a void in the shape of the cannula. The layer of acute inflammatory tissue varied in thickness, composition and continuity at different locations around the cannula. This layer was composed of compressed connective tissue/adipose cells, thrombus/fibrin, inflammatory cells, and freshly deposited collagen. The pathologists graded the histology: 7.0 mm insertion depth, 2.0 mm debris field, regular reservoir geometry, +3 fibrin deposition in reservoir, +1 reticulum fiber disruption, +1 collagen deposition at tip, +1 fat necrosis, +1 hemorrhage at tip, distorted blood vessel integrity at tip base, and regulator typography of tissue planes along path of cannula insertion. From left to right: H&E stain 20x magnification, Trichrome stain 40x magnification, and Reticulum stain 40x magnification. The cannula did not kink during/after insertion, did not produce an insulin pump occlusion alarm, and did not leak blue dye onto the skin on day 5.

**
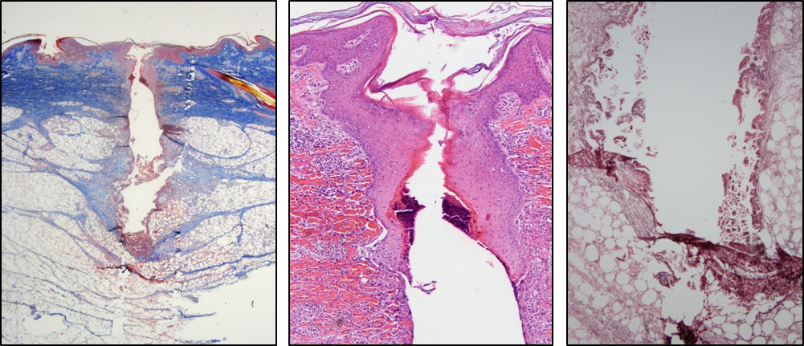
**

**Figure S6** – Swine 6, CC1: Commercial CSII catheter implanted within the subcutaneous tissue of Swine 6 infused with U-100 insulin lispro using a basal/bolus pattern for 5 days. The Teflon cannula was removed from the tissue after fixation- leaving a void in the shape of the cannula. The layer of acute inflammatory tissue varied in thickness, composition and continuity at different locations around the cannula. This layer was composed of compressed connective tissue/adipose cells, thrombus/fibrin, inflammatory cells, and freshly deposited collagen. Epidermis and dermis cells migrated > 1mm downward around the cannula shaft. The pathologists graded the histology: 6.8 mm insertion depth, 3.2 mm debris field, regular reservoir geometry, +3 fibrin deposition in reservoir, +1 reticulum fiber disruption, +2 collagen deposition at tip, +2 fat necrosis, +2 hemorrhage at tip, distorted blood vessel integrity at tip base, and regulator typography of tissue planes along path of cannula insertion. From left to right: Trichrome stain 20x magnification, H&E stain 40x magnification, and Reticulin stain 100x magnification. The cannula did not kink during/after insertion, did not leak blue dye onto the skin on day 5, but did produce an insulin pump occlusion alarm.


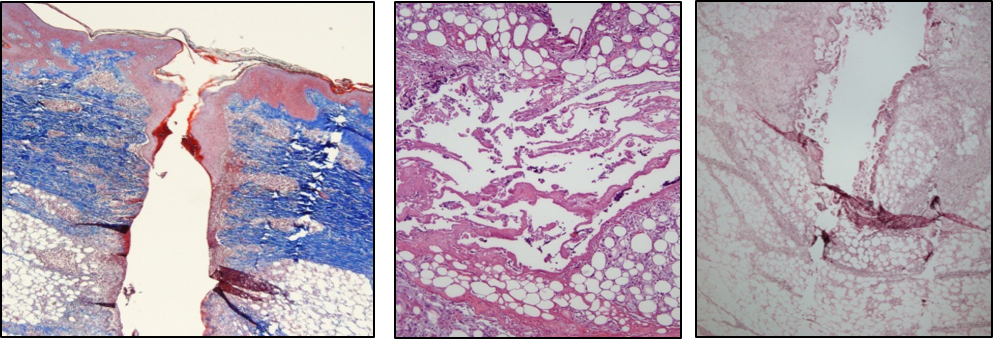


**Figure S7** – Swine 6, CC2 (see above Figure S4E): Commercial CSII catheter implanted within the subcutaneous tissue of Swine 5 and infused with U-100 insulin lispro using a basal/bolus pattern for 5 days. This commercial CSII catheter was used for the insulin PK study on day 5. The Teflon cannula was removed from the tissue after fixation- leaving a void in the shape of the cannula. Compared with healthy swine tissue, the connective tissue septa are thickened, distorted in a downward direction, and infiltrated with inflammatory cells. The layer of acute inflammatory tissue varied in thickness, composition and continuity at different locations around the cannula. Tissue surrounding the distal end of the cannula consisted of damaged connective tissue/adipose cells (debris), thrombus/fibrin and inflammatory cells. The pathologists graded the histology: 7.8 mm insertion depth, 2.2 mm debris field, regular reservoir geometry, +3 fibrin deposition in reservoir, +2 reticulum fiber disruption, +1 collagen deposition at tip, + 2 fat necrosis, + 2 hemorrhage at tip, distorted blood vessel integrity at tip base, and regulator typography of tissue planes along path of cannula insertion. From left to right: H&E stain 20x magnification, H&E stain 100x magnification near cannula tip and Reticulin stain 40x magnification.


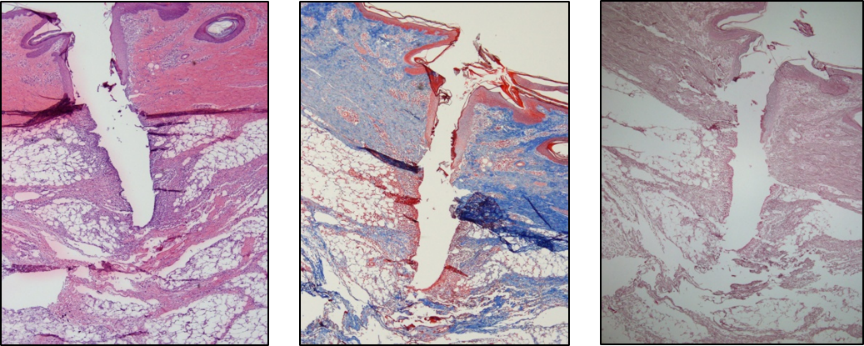


**Figure S8** – Swine 6, CC4: Commercial CSII catheter implanted within the subcutaneous tissue of Swine 6 infused with preservative free saline using a basal/bolus pattern for 5 days. The Teflon cannula was removed from the tissue after fixation- leaving a void in the shape of the cannula. Compared with healthy swine tissue, the connective tissue septa are thickened and infiltrated with inflammatory cells. The layer of acute inflammatory tissue was composed of compressed connective tissue/adipose cells, thrombus/fibrin, inflammatory cells, and freshly deposited collagen. This layer varied in thickness, composition and continuity at different locations around the cannula. Damaged cells and connective tissue not firmly attached to adjacent cells/tissue may have been lost during processing. The pathologists graded the histology: 6.2 mm insertion depth, 2.3 mm debris field, regular reservoir geometry, +2 fibrin deposition in reservoir, +1 reticulum fiber disruption, +1 collagen deposition at tip, +1 fat necrosis, +1 hemorrhage at tip, distorted blood vessel integrity at tip base, and intermittent typography of tissue planes along path of cannula insertion. From left to right: H&E stain 20x magnification, Trichrome stain 20x magnification, and Reticulum stain 20x magnification. The cannula did not produce an insulin pump occlusion alarm, was slightly kinked, and leaked a small amount of blue dye onto the skin surface during a 5 U bolus on day 5.


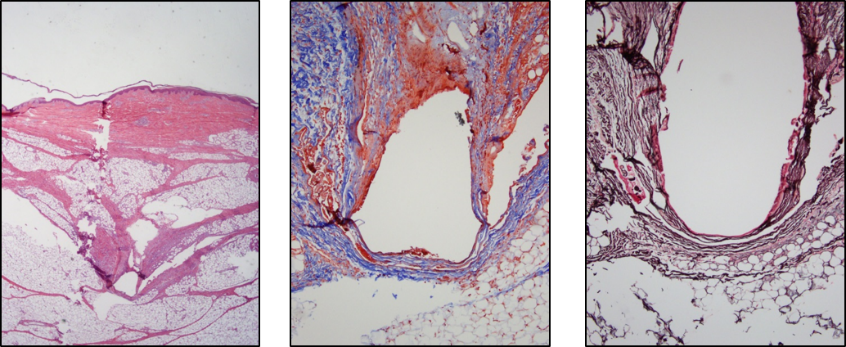


**Figure S9 –** Swine 6, CC6: Tissue histology surrounding a commercial CSII catheter filled with preservative free saline and capped (not infused); implanted within the subcutaneous tissue of Swine 6 for five days. The Teflon cannula was removed from the tissue after fixation- leaving a void in the shape of the cannula. Compared with healthy swine tissue, the connective tissue septa are thickened, distorted in a downward direction, and infiltrated with inflammatory cells. The surrounding layer was composed of compressed connective tissue/adipose cells, thrombus/fibrin, inflammatory cells, plasma and freshly deposited collagen/reticulin fibers. This layer of acute inflammatory tissue varied in thickness, composition and continuity at different locations around the cannula. The pathologists graded the histology: 6.6 mm insertion depth, 1.9 mm debris field, regular reservoir geometry, +1 fibrin deposition in reservoir, +1 reticulum fiber disruption, +1 collagen deposition at tip, + 2 fat necrosis, +2 hemorrhage at tip, intact blood vessel integrity at tip base, and regular typography of tissue planes along path of cannula insertion.From left to right: H&E stain 20x magnification (with artifact), Trichrome stain 40x magnification, and Reticulin stain 40x magnification. The cannula was not kinked but did produce an insulin pump occlusion alarm and leaked a small amount of blue dye onto the skin surface during a 5 U bolus on day 5.

**
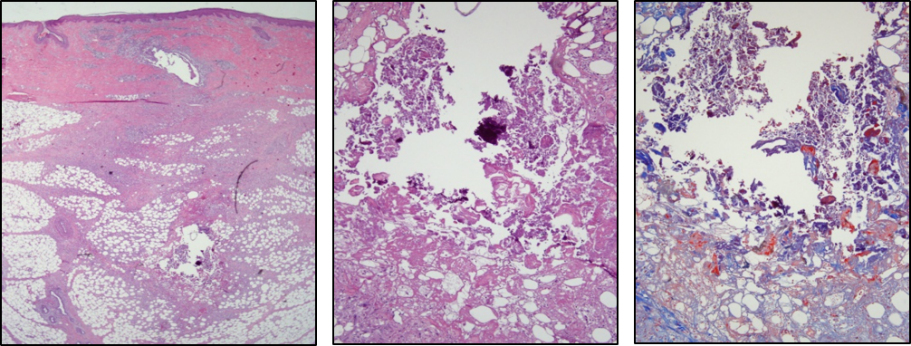
**

**Figure S10 –** Swine 5, IC1 (see above Figure S5): Investigational CSII catheter was implanted within the subcutaneous tissue of Swine 5 and infused with U-100 insulin lispro using a basal/bolus pattern for 5 days. This investigational CSII catheter was used for the insulin PK study on day 5. The cannula/needle was removed from the tissue after fixation- leaving a void in the tissue along the insertion path of the curved cannula. Compared with healthy swine tissue, the connective tissue septa are thickened and infiltrated with inflammatory cells. The layer of acute inflammatory tissue varied in thickness, composition and continuity at different locations around the cannula. The inflammatory layer was discontinuous and sparse at several locations. Tissue surrounding the distal end of the cannula/needle consisted of damaged connective tissue/adipose cells (debris), thrombus/fibrin and inflammatory cells. The pathologists graded the histology: 8.9 mm insertion depth, 3.8 mm debris field, irregular reservoir geometry, +1 fibrin deposition in reservoir, +2 reticulum fiber disruption, +1 collagen deposition at tip, +2 fat necrosis, +1 hemorrhage at tip, distorted blood vessel integrity at tip base, and irregular typography of tissue planes along path of cannula insertion. From left to right: H&E stain 20x magnification, H&E stain 100x magnification at cannula tip and Trichrome stain 100x magnification at cannula tip.


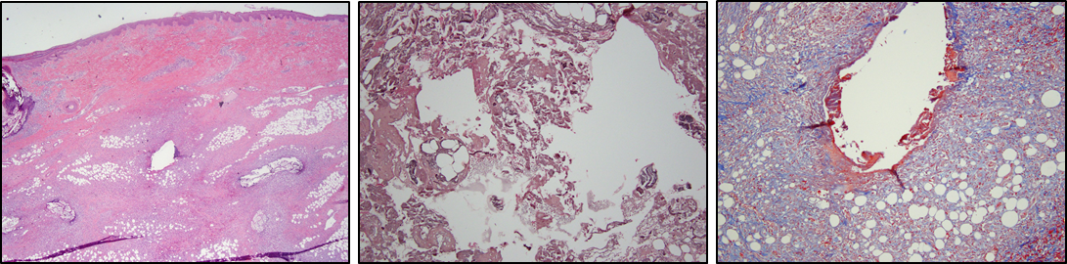


**Figure S11 –** Swine 5, IC2: Investigational CSII catheter implanted within the subcutaneous tissue of Swine 5 infused with U-100 insulin lispro using a basal/bolus pattern for 5 days. The cannula/needle was removed from the tissue after fixation- leaving a void in the tissue along the insertion path of the curved cannula. Compared with healthy swine tissue, the connective tissue septa are thickened and infiltrated with inflammatory cells. The layer of acute inflammatory tissue varied in thickness, composition and continuity at different locations around the cannula. Tissue surrounding the distal end of the cannula/needle consisted of damaged connective tissue/adipose cells (debris), thrombus/fibrin, plasma and inflammatory cells. The pathologists graded the histology: 9.2 mm insertion depth, 4.6 mm debris field, irregular reservoir geometry, +1 fibrin deposition in reservoir, +2 reticulum fiber disruption, +1 collagen deposition at tip, +3 fat necrosis, +2 hemorrhage at tip, disrupted blood vessel integrity at tip base, and irregular typography of tissue planes along path of cannula insertion. From left to right: H&E stain 20x magnification, Reticulin stain 100x magnification at cannula tip and Trichrome stain 100x magnification proximal to cannula tip. The cannula was not kinked, did not leak blue dye onto the skin surface during a 50 U bolus on day 5, but did produce an insulin pump occlusion alarm.


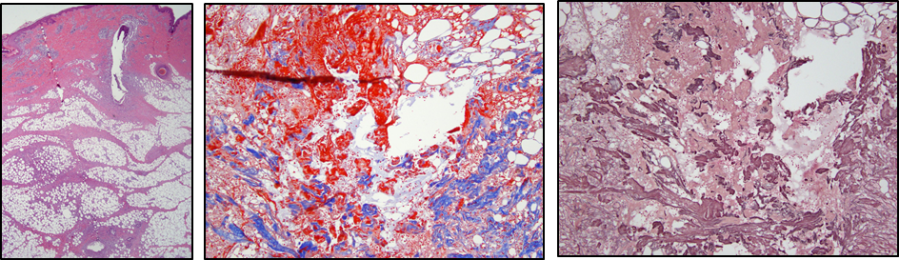


**Figure S12 –** Swine 5, IC4: Investigational CSII catheter implanted within the subcutaneous tissue of Swine 5 infused with preservative free saline using a basal/bolus pattern for 5 days. The cannula/needle was removed from the tissue after fixation- leaving a void in the tissue along the insertion path of the curved cannula. Compared with healthy swine tissue, the connective tissue septa are thickened and infiltrated with inflammatory cells. The layer of acute inflammatory tissue varied in thickness, composition and continuity at different locations around the cannula. Tissue surrounding the distal end of the cannula/needle consisted of damaged connective tissue/adipose cells (debris), thrombus/fibrin, plasma and inflammatory cells. The pathologists graded the histology: 8.0 mm insertion depth, 2.8 mm debris field, regular reservoir geometry, +2 fibrin deposition in reservoir, +1 reticulum fiber disruption, +2 collagen deposition at tip, +1 fat necrosis, +1 hemorrhage at tip, distorted blood vessel integrity at tip base, and intermittent typography of tissue planes along path of cannula insertion. From left to right: H&E stain 20x magnification, Trichrome stain 100x magnification at cannula tip and Reticulin stain 100x magnification at cannula tip. The cannula was not kinked but did produce an insulin pump occlusion alarm and did leak a small amount of blue dye onto the skin surface during a 5 U bolus on day 5.

**
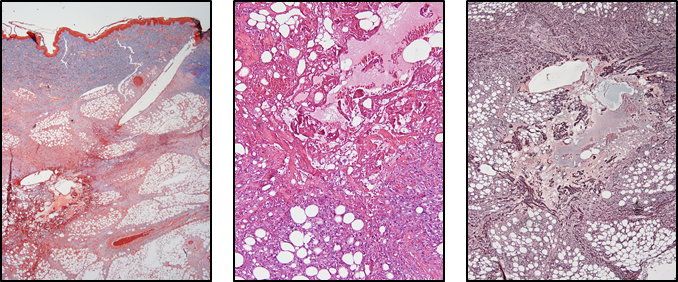
**

**Figure S13 –** Swine 6, IC1 (see above Figure S5A): The investigational CSII catheter was implanted within the subcutaneous tissue of Swine 6 and infused with U-100 insulin lispro using a basal/bolus pattern for 5 days. This investigational CSII catheter was used for the insulin PK study on day 5. The cannula/needle was removed from the tissue after fixation- leaving a void in the tissue along the insertion path of the curved cannula. Compared with healthy swine tissue, the connective tissue septa are thickened and infiltrated with inflammatory cells. The layer of acute inflammatory tissue varied in thickness, composition and continuity at different locations around the cannula. Tissue surrounding the distal end of the cannula/needle consisted of damaged connective tissue/adipose cells (debris), thrombus/fibrin, plasma and inflammatory cells. The pathologists graded the histology: 6.5 mm insertion depth, 4.1 mm debris field, irregular reservoir geometry, +3 fibrin deposition in reservoir, +3 reticulum fiber disruption, 0 collagen deposition at tip, + 3 fat necrosis, + 2 hemorrhage at tip, disrupted blood vessel integrity at tip base, and irregular typography of tissue planes along path of cannula insertion. From left to right: Trichrome stain 20x magnification, H&E stain 100x magnification at cannula tip and Reticulin stain 40x magnification at cannula tip.


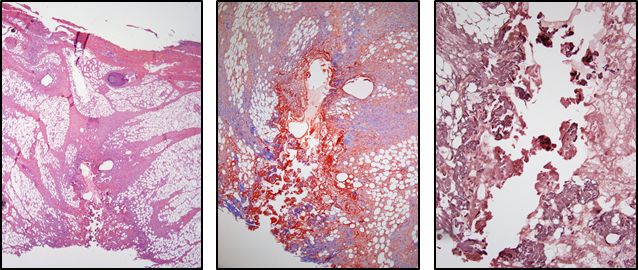


**Figure S14 –** Swine 6, IC2: Investigational CSII catheter implanted within the subcutaneous tissue of Swine 6 infused with U-100 insulin lispro using a basal/bolus pattern for 5 days. The cannula/needle was removed from the tissue after fixation- leaving a void in the tissue along the insertion path of the curved cannula. Compared with healthy swine tissue, the connective tissue septa are thickened and infiltrated with inflammatory cells. The layer of acute inflammatory tissue varied in thickness, composition and continuity at different locations around the cannula. Tissue surrounding the distal end of the cannula/needle consisted of damaged connective tissue/adipose cells (debris), thrombus/fibrin, plasma and inflammatory cells. The pathologists graded the histology: 8.2 mm insertion depth, 3.9 mm debris field, irregular reservoir geometry, +3 fibrin deposition in reservoir, +3 reticulum fiber disruption, +2 collagen deposition at tip, +3 fat necrosis, +2 hemorrhage at tip, disrupted blood vessel integrity at tip base, and intermediate typography of tissue planes along path of cannula insertion. From left to right: H&E stain 20x magnification, Trichrome stain 40x magnification at cannula tip and Reticulin stain 100x magnification at cannula tip. The cannula was not kinked and did not leak blue dye onto the skin surface during a 50 U bolus on day 5; but did produce an insulin pump occlusion alarm.


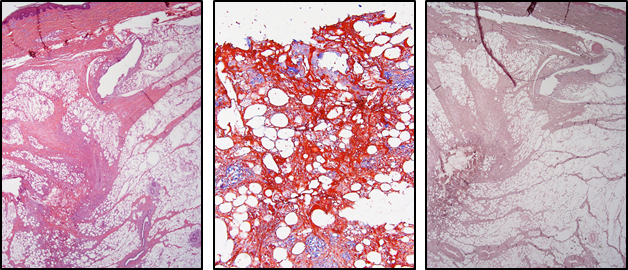


**Figure S15 –** Swine 6, IC6: Tissue histology surrounding an Investigational CSII catheter filled with preservative free saline and capped; implanted within the subcutaneous tissue of Swine 6 for five days. The cannula/needle was removed from the tissue after fixation- leaving a void in the tissue along the insertion path of the curved cannula. Compared with healthy swine tissue, the connective tissue septa are thickened and infiltrated with inflammatory cells. The layer of acute inflammatory tissue varied in thickness, composition and continuity at different locations around the cannula. Tissue surrounding the distal end of the cannula/needle consisted of damaged connective tissue/adipose cells (debris), thrombus/fibrin, plasma and inflammatory cells. The pathologists graded the histology: 8.0 mm insertion depth, 4.1 mm debris field, irregular reservoir geometry, +3 fibrin deposition in reservoir, +3 reticulum fiber disruption, +2 collagen deposition at tip, + 3 fat necrosis, + 2 hemorrhage at tip, disrupted blood vessel integrity at tip base, and irregular typography of tissue planes along path of cannula insertion. From left to right: H&E stain 20x magnification, Trichrome stain 100x magnification and Reticulin stain 20x magnification at cannula tip. The cannula was not kinked and did not produce an insulin pump occlusion alarm; but did leak a large amount of blue dye onto the skin surface during a 5 U bolus on day 5.


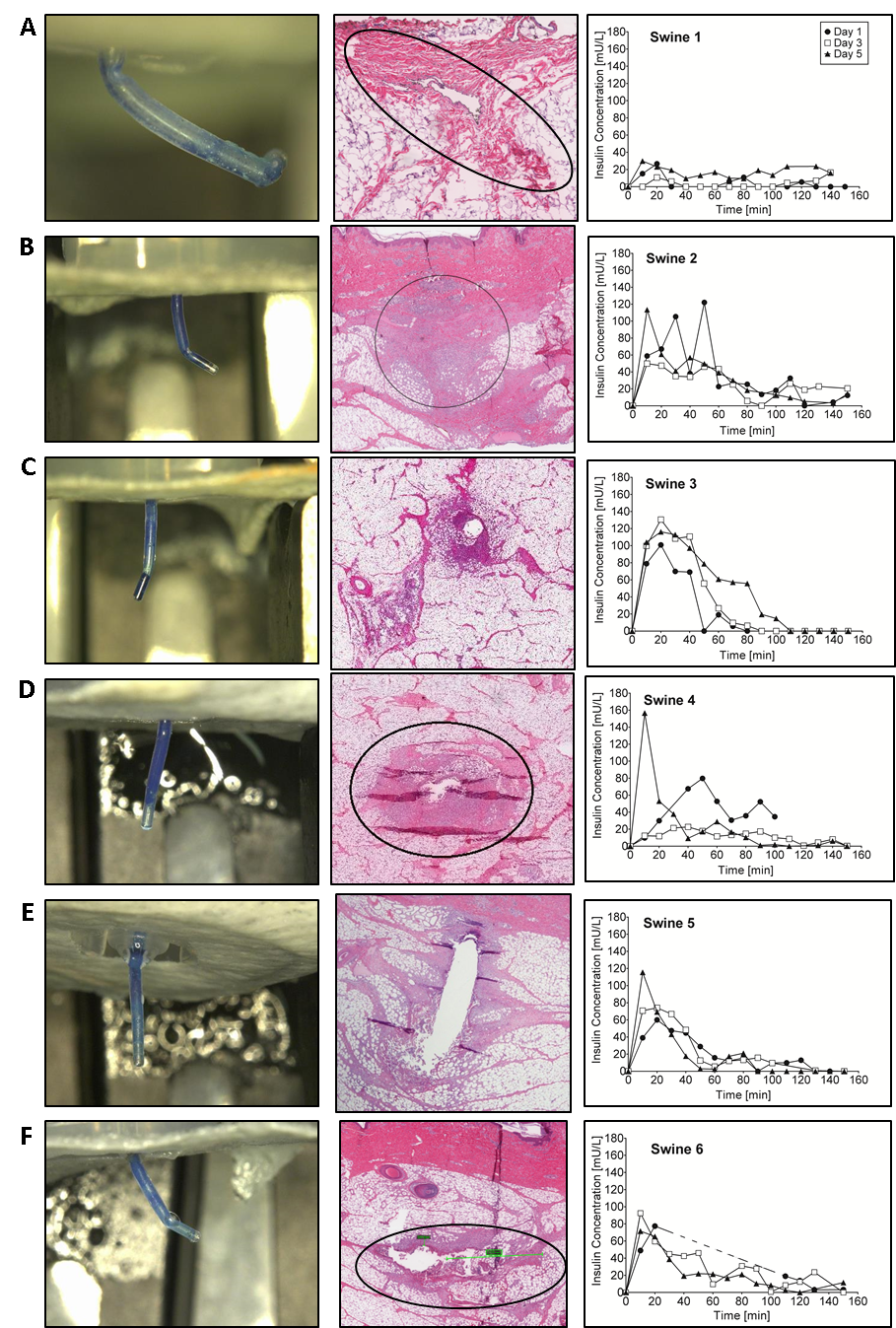


**Figure S16:** Images of commercial catheters (CC) used for PK studies, the corresponding tissue histology (H&E, 20x) and insulin PK curves from days 1, 3 and 5 from each swine. Tissue specimens were excised on day 5 after implantation. The clear white void in each histology photo was caused by the loss of damaged cells, connective tissue and extra-cellular matrix following fixation and removal of the CSII cannula.

***A****:* Swine 1- Insertion of CC2 caused the cannula to kink within the superficial subcutaneous tissue, did not produce a pump occlusion alarm, did not leak dye/insulin into the CSII hub, but did leak a large amount onto the skin surface. The distal end of the CSII cannula was partially surrounded by 1-3 mm layer of acute inflammatory tissue. Insulin absorption from the CC into the circulation on days 1, 2 and 3 was very limited.

***B****:* Swine 2- The CC1 cannula was fully inserted within the subcutaneous tissue, developed a moderate kink, did not produce a pump occlusion alarm, did not leak dye/insulin onto the skin surface, but did have a mild leak into the CSII hub. A 2-3 mm layer of acute inflammatory tissue surrounded the cannula (circle), most pronounced near the cannula tip (below and right of circle). Subcutaneous tissue was forced downward and compressed due to cannula insertion (below circle). The pathologists graded the histology: 3.1 mm debris field, regular reservoir geometry, +2 fibrin deposition in reservoir, +1 reticulum fiber disruption, +1 collagen deposition at tip, +1 fat necrosis, regulator typography of tissue planes along path of cannula insertion. Insulin absorption from the CC into the circulation on days 1, 3 and 5 was average with high PK variability.

***C****:* Swine 3- The CC2 cannula was fully inserted within the subcutaneous tissue, did not kink, did not leak insulin/dye onto the skin surface, did not have leak into the CSII hub and did not produce a pump occlusion alarm. The layer of acute inflammatory tissue that surrounded the cannula was incomplete with regions of low density on the cross-section image. The pathologists graded the histology: 3.3 mm debris field, regular reservoir geometry, +3 fibrin deposition in reservoir, +1 reticulum fiber disruption, +1 collagen deposition at tip, +1 fat necrosis, regulator typography of tissue planes along path of cannula insertion. Insulin absorption from the CC into the circulation on days 1, 3 and 5 was average with low PK variability.

***D*:** Swine 4- The CC cannula was fully inserted within the subcutaneous tissue, developed a slight kink, did not leak insulin/dye onto the skin surface, did not have leak into the CSII hub, and did not produce a pump occlusion alarm. The 2-3 mm thick layer of acute inflammatory tissue that surrounded the cannula was incomplete with regions of low density on the cross-section image. The pathologists graded the histology: 1.9 mm debris field, regular reservoir geometry, +3 fibrin deposition in reservoir, +1 reticulum fiber disruption, +1 collagen deposition at tip, +2 fat necrosis, regulator typography of tissue planes along path of cannula insertion. Insulin absorption from the CC into the circulation on days 1, 3 and 5 was average with high PK variability.

***E***: Swine 5- The CC2 cannula was fully inserted within the subcutaneous tissue, did not kink, did not leak insulin/dye onto the skin surface, did not have leak into the CSII hub, and did not produce a pump occlusion alarm. The 2-3 mm thick layer of acute inflammatory tissue that surrounded the cannula was complete and dense along the shaft but incomplete with regions of low tissue density near the cannula tip. The pathologists graded the histology: 2.2 mm debris field, regular reservoir geometry, +3 fibrin deposition in reservoir, +2 reticulum fiber disruption, +1 collagen deposition at tip, +2 fat necrosis, regulator typography of tissue planes along path of cannula insertion. Insulin absorption from the CC into the circulation on days 1, 3 and 5 was average with high PK variability.

***F****:* Swine 6- The CC2 cannula was fully inserted within the subcutaneous tissue, had a moderate kink, did not produce a pump occlusion alarm, did not leak insulin/dye onto the skin surface, but did have a small leak into the CSII hub. The 2-3 mm thick layer of acute inflammatory tissue that surrounded the cannula was incomplete with regions of low tissue density. The pathologists graded the histology: 2.6 mm debris field, irregular reservoir geometry, +3 fibrin deposition in reservoir, +2 reticulum fiber disruption, +1 collagen deposition at tip, +2 fat necrosis, intermittent typography of tissue planes along path of cannula insertion. Insulin absorption from the CC into the circulation on days 1, 3 and 5 was lower than average with low PK variability.

**
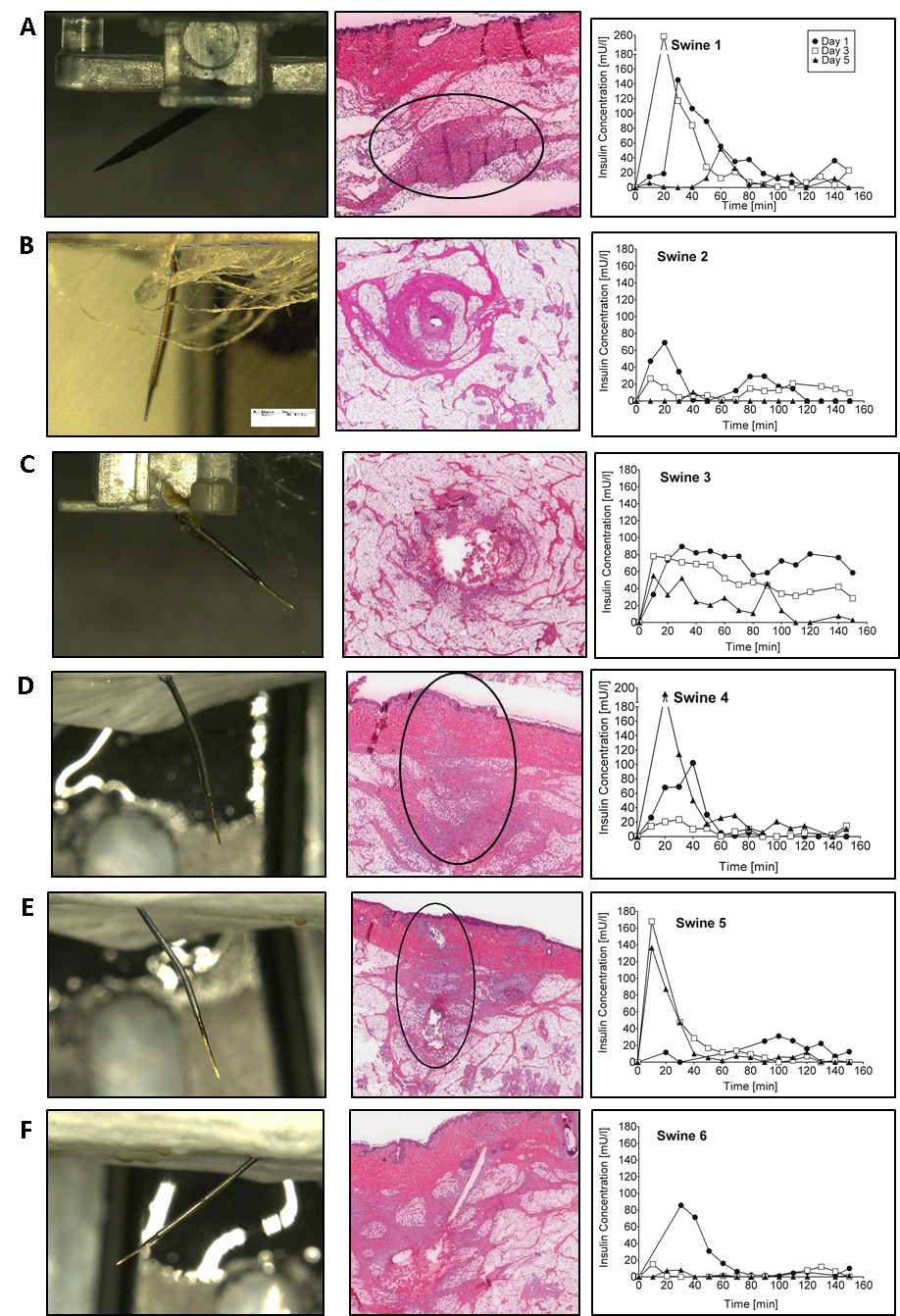
**

**Figure S17** – Images of investigational catheters (IC) used for PK studies, the corresponding tissue histology (H&E, 20x) and insulin PK curves from days 1, 3 and 5 from each swine. Tissue specimens were excised on day 5 after implantation. The clear white void in each histology photo was caused by the loss of damaged cells, connective tissue and extra-cellular matrix following fixation and removal of the CSII cannula. The subcutaneous tissue surrounding the cannula tip had the most severe damage.

***A:*** Swine 1- The IC1 cannula was fully inserted within the subcutaneous tissue at a 45 degree angle, did not kink, did not leak dye/insulin onto the skin surface, did produce a pump occlusion alarm (day 3 only) and did have a small amount leakage into the CSII hub. The 3-6 mm thick layer of acute inflammatory tissue that surrounded the cannula was nearly complete with regions of high and low tissue density. The pathologists graded the histology: 7.9 mm debris field, irregular reservoir geometry, +1 fibrin deposition in reservoir, +3 reticulum fiber disruption, +1 collagen deposition at tip, +3 fat necrosis, irregular typography of tissue planes along path of cannula insertion. Insulin absorption from the IC into the circulation was high on days 1 and 3 and low/delayed on day 5 with high PK variability.

***B:*** Swine 2- The IC2 cannula was fully inserted within the subcutaneous tissue at a 80 degree angle, did not kink, did not leak dye/insulin into the hub, did not produce several pump occlusion alarms, and did leak a large amount onto the skin surface. The 2-4 mm thick layer of acute inflammatory tissue that surrounded the cannula was nearly complete with regions of high and low tissue density. The pathologists graded the histology: 3.2 mm debris field, regular reservoir geometry, +2 fibrin deposition in reservoir, +2 reticulum fiber disruption, +1 collagen deposition at tip, +2 fat necrosis, intermittent typography of tissue planes along path of cannula insertion. Insulin absorption from the IC into the circulation was low on day 1 and limited on days 3 and day 5 with high PK variability.

***C:*** Swine 3- The IC cannula was fully inserted within the subcutaneous tissue at a 45 degree angle, did not kink, did not leak dye/insulin onto the skin surface, did produce a pump occlusion alarm (day 5 only) and did leak a small amount into the CSII hub. The layer of acute inflammatory tissue that surrounded the cannula was incomplete with regions of low tissue density on the cross-section image. The pathologists graded the histology: 6.8 mm debris field, irregular reservoir geometry, +2 fibrin deposition in reservoir, +3 reticulum fiber disruption, +1 collagen deposition at tip, +2 fat necrosis, intermittent typography of tissue planes along path of cannula insertion. Insulin absorption from the IC into the circulation was average on days 1 and 5 and limited on day 5 with high PK variability.

**D:** Swine 4- The IC2 cannula was fully inserted within the subcutaneous tissue at a 80 degree angle, developed a slight kink, did not leak insulin/dye onto the skin surface, did not leak into the CSII hub, and did produce a pump occlusion alarm (day 5 only). The 2-3 mm thick layer of acute inflammatory tissue that surrounded the cannula was incomplete with regions of low density on the cross-section image. The pathologists graded the histology: 4.3 mm debris field, irregular reservoir geometry, +2 fibrin deposition in reservoir, +3 reticulum fiber disruption, +1 collagen deposition at tip, +3 fat necrosis, intermittent typography of tissue planes along path of cannula insertion. Insulin absorption from the IC into the circulation was average/delayed on day 1, limited on day 3 and high on day 5 with high PK variability.

**E:** Swine 5- The IC1 cannula was fully inserted within the subcutaneous tissue at a 60 degree angle, did not kink, did not leak insulin/dye onto the skin surface, did not leak into the CSII hub, and did not produce a pump occlusion alarm. The 2-3 mm thick layer of acute inflammatory tissue that surrounded the cannula was complete and dense along the shaft but incomplete with regions of low density near the cannula tip. The pathologists graded the histology: 3.8 mm debris field, irregular reservoir geometry, +1 fibrin deposition in reservoir, +2 reticulum fiber disruption, +1 collagen deposition at tip, +2 fat necrosis, irregular typography of tissue planes along path of cannula insertion. Insulin absorption from the IC into the circulation was limited on day 1 and high on days 3 and 5 with high PK variability.

**F:** Swine 6- The IC1 cannula was fully inserted within the subcutaneous tissue at a 45 degree angle, did not kink, did not leak insulin/dye onto the skin surface, did not produce a pump occlusion alarm, but did leak a moderate amount into the CSII hub. The 2-8 mm thick layer of acute inflammatory tissue that surrounded the cannula was incomplete with regions of low density. The pathologists graded the histology: 4.1 mm debris field, irregular reservoir geometry, +3 fibrin deposition in reservoir, +3 reticulum fiber disruption, 0 collagen deposition at tip, +3 fat necrosis, irregular typography of tissue planes along path of cannula insertion. Insulin absorption from the IC into the circulation was average on day 1 and limited on days 3 and 5 with high PK variability. Images of investigational catheters used for PK studies after removal and the corresponding histology (H&E, 20x) and PK curves of each swine. Note: The ideal insertion angle for this catheter is 60°. ***A:*** Swine 1. Inflamed adipose tissue around the cannula’s tip indicated by circle; the debris field is 8 mm wide. Insulin absorption is adequate on days 1 and 3 but fails on day 5 of wear time. ***B:*** Swine 2. Catheter did not insert adequately (insertion angle 102°) which may explain the low insulin absorption throughout wear time. Histology showed moderate (+2) tissue disruption, necrosis and fibrin deposition (*image not available*). ***C:*** Swine 3. Catheter inserted in appropriate angle but PK did not produce C_max_ peak on either of the days. Tissue sectioned in “bull’s eye perspective’ parallel to skin. Image shows a tissue debris field of 7 mm around void. Reticulin fiber disruption was graded severe (+3) by pathologist. ***D:*** Swine 4. Catheter bent upon insertion. Insulin absorption failed on day 3 but recovered by day 5 of wear time. Circle in histology picture marks inflamed area along the path of insertion where tissue planes were pushed down by the catheter. ***E:*** Swine 5. There was no insulin absorption on day 1 but high and adequate absorption on days 3 and 5. Histology section shows two voids left by bent catheter and disrupted tissue around the cannula’s sharp tip. ***F:*** Swine 6. Catheter inserted in appropriate angle but produced adequate PK only on day 1 and none on days 3 and 5. Angled insertion channel visible in H&E section. Catheter caused severe (+3) reticular fiber disruption, fibrin deposition and fat necrosis.
